# Association between Sports Participation in Early Life and Arterial Intima-Media Thickness among Adults

**DOI:** 10.3390/medicina54050085

**Published:** 2018-11-13

**Authors:** André O. Werneck, Manoel C. S. Lima, Ricardo R. Agostinete, Danilo R. Silva, Bruna C. Turi-Lynch, Jamile S. Codogno, Rômulo A. Fernandes

**Affiliations:** 1 Laboratory of InVestigation in Exercise (LIVE), Department of Physical Education, Sao Paulo State University (UNESP), Presidente Prudente 19060-900, Brazil; mcspiguelima@hotmail.com (M.C.S.L.); ricardoagostinete@gmail.com (R.R.A.); brunatlynch@gmail.com (B.C.T.-L.); jamile@fct.unesp.br (J.S.C.); romulo.a.fernandes@unesp.br (R.A.F.); 2 Department of Physical Education, Federal University of Sergipe—UFS, São Cristóvão 49100-000, Brazil; danilorpsilva@gmail.com

**Keywords:** atherosclerosis, sports medicine, motor activity, adolescence, cardiovascular diseases

## Abstract

*Background:* Early sports practice is associated with several health benefits during childhood and adolescence, moreover, recent evidence also suggests that sports during childhood and adolescence can produce some benefits during adulthood. However, the association between early sports practice and arterial thickness is not clear. Thus, our aim was analyze the association between sports participation in childhood and adolescence, carotid/femoral intima–media thickness, and blood flow index in adulthood. *Material and Methods:* Sample was composed of 107 adults (64 males) between 30 years and 50 years, which were recruited from different gyms and university staff from São Paulo State University. Participants were divided according to sports participation in early life (engaged in sports during childhood and adolescence (*n* = 52) and no engagement in sports during childhood and adolescence (*n* = 55)). Carotid and femoral intima–media thickness were measured through Doppler ultrasonography method. Carotid and femoral index were estimated from ultrasonography measures. As covariates, the following were adopted: chronological age, sex, body fat (through dual-energy x-ray absorptiometry), c-reactive protein, HOMA, alcohol consumption, tobacco smoking, mean arterial pressure and current physical activity (pedometer). General estimating equations were used, adopting *p* < 0.05. *Results:* In the adjusted analyses, early sports participation was associated with lower carotid intima–media index (early sports participation: 0.64 mm ± 0.14 mm vs. no early sports participation: 0.71 mm ± 0.21 mm; *p* = 0.011), but not associated with femoral intima–media thickness, carotid resistive index and femoral resistive index after the adjustment by potential confounders. *Conclusions:* Sports participation in childhood and adolescence was associated with a reduced carotid intima–media thickness, independently of relevant confounders.

## 1. Background

Over the last decades, chronic diseases became one of the greatest causes of premature death worldwide [1]. Cardiovascular diseases receive special attention among chronic diseases due to its burden on mortality. For instance, atherosclerosis is one of the most significant causes of death [1], while atheroma plaque formation also increases the mortality risk without necessarily an atherosclerosis formation [2].

Carotid intima–media thickness (CIMT) is an independent factor for several cardiovascular diseases [3], given its association with atherosclerosis, correlation with atheroma plaque formation [4] and early mortality [5]. Therefore, arterial intima–media thickness (IMT) represents an important indicator for cardiovascular diseases screening [3,4].

In fact, not just biological (e.g., aging, genetics), but also behavioral variables have a relevant impact on IMT, such diet, smoking, sedentary behavior, physical activity and others [6,7,8]. Among these behaviors (also called non-pharmacological), regular engagement in exercise is associated with lower CIMT [9], and physical activity in adulthood is traditionally recognized to be a protective factor for cardiovascular diseases [10]. However, the effects of physical activity in early life (performed during childhood and adolescence) on adulthood health is unclear [11]. Recent evidence has shown that physical activity in early life, especially through sports participation, can protect against negative cardiovascular outcomes (arterial thickening and arterial hypertension) in adulthood through different paths, such as tracking of physical activity and adiposity, as well as DNA methylation [12,13].

However, previous investigations failed to present a broader range of possible confounders, as glycemic metabolism indicators, inflammatory markers, body adiposity, objectively measured physical activity, and blood pressure, which are potential mediators of the arterial thickening during adulthood [9,14,15,16]. Moreover, the association between physical activity performed in early life and arterial blood flow indexes remains unclear. Thus, we aimed to analyze the association between sports participation in early life (childhood and adolescence) with the carotid/femoral intima–media thickness, and blood flow index in adulthood.

## 2. Material and Methods

This cross-sectional study was composed of 122 adults (53 females), between 30 and 50 years, conducted in Presidente Prudente, São Paulo, Brazil during 2013. The sample was recruited from fitness clubs of the city and São Paulo State University (fitness clubs, *n* = 100; university staff, *n* = 22). Sample size was based on an equation for correlation, which adopted *r* = 0.26, with a power of 80% and statistical significance of 5%.

The inclusion criteria to be eligible were: (i) either sports participation in early life (both childhood and adolescence) or absence of sports participation in early life (both childhood and adolescence); (ii) aged between 30 and 50; (iii) no previous history of stroke or infarction; (iv) no amputation or visual impairment due to diabetes mellitus. Due to missing data, 15 (5 males) subjects were excluded from the sample. Thus, the final sample was composed of 107 adults (64 males). All participants agreed to participate and signed a consent form. All procedures were approved in the Ethics Research Committee of the São Paulo State University. More detailed procedures were previous published [17].

Sports participation in childhood and adolescence was assessed by two questions: (1) “Outside of school, did you engage in any organized/supervised sport during at least one year between seven and ten years old?”; and (2) “Outside of school, did you engage in any organized/supervised sport during at least one year between 11 and 17 years old?”. In this way, only the volunteers who answered “yes” or “no” on both questions were selected to participate in the study (the questions have good reproducibility, previously tested in [18]).

A trained physician measured resistive index, carotid and femoral intima–media thickness (CIT and FIT, respectively) using a Doppler ultrasound device (Toshiba Xario, SSA-660A, Tokyo, Japan) adopting standardized protocols [19]. Before the exam (all measurements were carried out between 08 and 11 a.m.), all subjects were at rest, lying in a supine position in a quiet, acclimatized room. Measurement of the posterior wall of the carotid artery was performed with neck in hyperextension and slightly inclined at 45°. Three measurements of each artery were taken (stretch of 15 mm free of plaques) and mean values were expressed as millimeters (mm). The ultrasound’s software estimated carotid and femoral resistive indexes (markers of blood flow).

Current physical activity level (Current-PA) was measured by pedometer (Digi- Walker Yamax, SW200, Tokyo, Japan) and expressed as average steps of seven consecutive days wearing the device.

Current smoking (yes or no) and alcohol consumption (weekly consumption) were assessed through an interview. The measurement of resting blood pressure was performed three times with an interval of one minute between each measurement through auscultatory method (systolic (SBP) and diastolic blood pressure (DBP)) were measured with the assistance of aneroid sphygmomanometer (Bic^®^) and a stethoscope (Littmann Classic II^®^) [20]. Blood pressure was the average of the last two measurements, while mean blood pressure was estimated through the formula: Mean Arterial Pressure = 1/3(SBP) + 2/3(DBP).

A private laboratory collected blood samples after 12 h fasting and metabolic variables (fasting glucose and insulin) and inflammatory markers (highly sensitive C-reactive protein [hsCRP]) were measured. Homeostatic Model Assessment-Insulin resistance (HOMA-IR) was estimated using the formula: HOMA-IR = (Fasting glucose × insulin) ÷ 22.5 [21].

A trained researcher estimated body fatness (BF in percentage) using a Dual Energy X-ray Absorptiometry device (DXA) (Lunar DPX-NT; General Electric Healthcare, Little Chalfont, Buckinghamshire, UK) and a GE Medical System Lunar software (version 4.7, GE Healthcare, Chicago, IL, EUA) in an university laboratory (temperature-controlled and with no metal close the participant).

Descriptive statistics were composed of median values and its range. Normality was tested using the Kolmogorov–Smirnov test. The Mann–Whitney test and chi-square test were used to compare groups. Covariance analysis (ANCOVA) was used to compare groups, with and without sports participation in early life, adjusting by covariates. Statistical significance (*p*) was set at values < 0.05. STATA (version 15.1, StataCorp, College Station, TX, USA) was the statistical software used for all analysis.

## 3. Results

The sample was composed of 107 adults (64 men), with a mean age of 39.6 years ± 3.6 years. Characteristics of the sample are presented in Table 1. Males presented greater BMI, SBP, DBP, mean blood pressure, CIT, FIT and carotid resistive index (*p* < 0.05) than females. On the other hand, females presented greater body fatness and femoral resistive index (*p* < 0.05).

**Figure 1:**
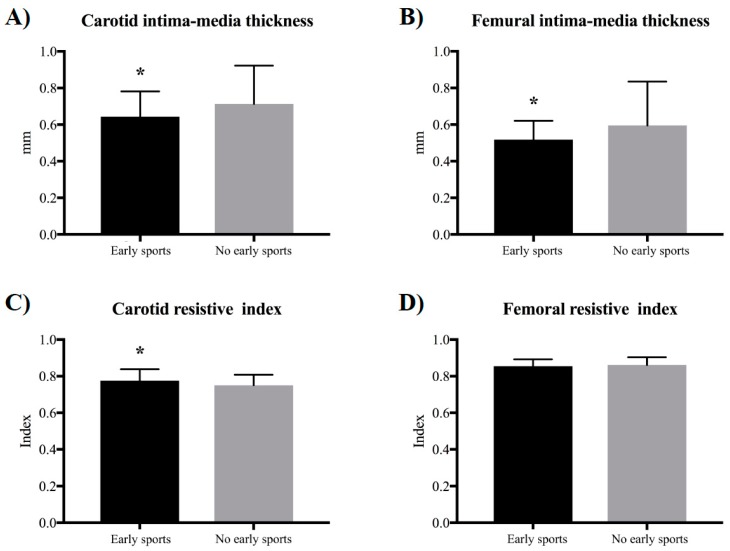
Carotid and femoral arterial thickness and resistance index according to sports participation in early life and No early sports participation group. Note. * *p* < 0.05 of ANCOVA (covariance analysis) adjusted by sex and chronological age. “Early sports” refers to early sports participation group and “no early sports” refers to the group without early sports participation.

Covariance analyses estimating the influence of sports participation in arterial thickness and resistive index adjusted by sex and chronological age are presented in Figure 1. Subjects who were engaged in sports in early life presented lower CIT (*p*-value = 0.014) and FIT (*p*-value = 0.012) when compared to adults not engaged in sports during childhood and adolescence, while carotid resistive index (*p*-value = 0.021) was higher in adults who were engaged in sports than the not engaged peers.

After the first set of analyses, we adjusted the models for potential mediators of the association between early sports participation and arterial thickness/resistive index at adulthood (i.e., current physical activity, tobacco smoking, alcohol consumption, body fat, C-reactive protein, HOMA index and mean blood pressure) (Table 2). After the adjustments, only CIT remained statistically related to sports participation in early life (*p* = 0.011).

## 4. Discussion

A greater arterial intima–media thickness is a recognized risk factor for mortality [22] and can be impacted by lifestyle indicators [9,23]. Here, we aimed to analyze the association between sports participation during childhood and adolescence and arterial outcomes (thickness and resistive index) during adulthood. Regardless of age and sex, we found that adults engaged in sports in childhood and adolescence presented lower CIT and FIT and a higher carotid resistive index. On the other hand, when adjusted by potential mediators (habitual physical activity, tobacco smoking, alcohol consumption, body fat, C-reactive protein, HOMA index and mean blood pressure), sports participation in early life was only associated with lower CIT.

Although femoral intima-media thickness and carotid resistive index were significantly adjusted by sex and chronological age, in the second set of analyses they were no longer significant, indicating that the effects of sports participation in early life on FIT and carotid resistive index were possibly mediated by other variables, such as inflammation, current physical activity, and obesity. Similarly, cross-sectional and longitudinal studies have reported the burden of sports participation on femoral artery structure modifications in pediatric groups [24] in which inflammation and body fatness seem to play an important role in this phenomenon.

Sports participation in early life has been recognized as a protective factor for chronic diseases also in adulthood [18], and it is believed that this protective effect happens due to the maintenance of lower body composition, higher physical activity levels and physical fitness throughout life [11,12]. In fact, the only consistent evidence supporting a direct effect of early sports participation on adult’s health is observed in bone tissue [25]. However, recent studies have argued about the existence of a possible direct effect on other health outcomes in adulthood [18], which would happen independent of current physical activity, body fatness and metabolic markers.

In this sense, we found that early sports participation was associated with a reduced CIT even after the adjustment by current physical activity, tobacco smoking, alcohol consumption, body fatness, C-reactive protein, HOMA index and mean blood pressure. Therefore, although other factors should be examined for CIT explanation (given an R^2^ of 0.053), we corroborate an alternative hypothesis of a possible direct effect of early sports participation on cardiovascular risk [12]. The pathway to support this direct effect is unclear, but it is postulated that in critical periods, like childhood and adolescence, the stress generated by sports participation can stimulate DNA methylation [26], leading to protection for chronic diseases independent of current lifestyle and/or biological conditions in adulthood.

Pälve et al. [13] found that physical activity during early adolescence was associated with carotid elasticity during adulthood, even after controlling for several covariates. Thus, our study adds due to the adjustment for an inflammatory variable (C-reactive protein) as well as for considering sports participation during childhood. Similarly, results from the Amsterdam Growth and Health Longitudinal Study presented an association between physical activity during adolescence and carotid thickness during adulthood [27], but the study did not consider potential mediators as body fat during adulthood, as well as lipid and inflammatory variables.

Our study has the strength to consider a greater number of potential confounders not previously controlled simultaneously in other studies [9,14,23,28]. Moreover, we have measured body fatness through an accurate method. However, our findings should be inferred with caution due to some limitations: (i) the reduced sample does not allow us to stratify by sex; (ii) retrospective design, even with good reproducibility [19], is exposed to recall bias and considers only a few points in the participants’ life; (iii) although the pedometer is a widely accepted method for measuring physical activity, it considers only steps and does not measure levels of physical activity [29]; (iv) we did not present an indicator of physical fitness, which is a potential mediator in the associations tested.

## 5. Conclusions

In summary, sports participation in early life was associated with reduced carotid intima-media thickness, independently of relevant confounders. These findings highlight the relevance of physical activity promotion in children and adolescents, as well as the central role that sports participation can assume in campaigns for physical activity promotion.

## Figures and Tables

**Table 1:** Characteristics of sample stratified by practice and non-practice of sports during childhood and adolescence (*n* = 107).

Variables	Early Sports Practice (*n* = 52)	No early Sports Practice (*n* = 55)	*p*-Value
Chronological age (years)	39.8 (29.7–50.5)	37.4 (29.6–53.7)	0.609
Body Mass (kg)	77.3 (45.9–103.1)	80.6 (51.4–137.3)	0.035
Stature (cm)	175.1 (150.1–195.0)	170.5 (148.6–188.0)	0.013
BMI (kg/m^2^)	24.3 (19.6–32.3)	28.1 (18.8–38.9)	<0.001
Body fat (%)	23.9 (7.9–50.0)	36.8 (20.1–57.8)	<0.001
Systolic blood pressure (mmHg)	110.0 (90.0–140.0)	120.0 (80.0–140.0)	0.141
Diastolic blood pressure (mmHg)	80.0 (60.0–90.0)	80.0 (60.0–90.0)	0.072
Mean blood pressure (score)	90.0 (71.7–108.3)	93.3 (66.7–106.7)	0.073
C-reactive protein (mg/dL)	0.87 (0.15–18.65)	2.49 (0.29–16.45)	<0.001
HOMA (score)	0.91 (0.21–3.21)	1.53 (0.51–8.40)	<0.001
Carotid intima-media thickness (mm)	0.57 (0.42–0.98)	0.65 (0.47–1.70)	0.057
Femoral intima-media thickness (mm)	0.49 (0.35–0.82)	0.55 (0.34–1.85)	0.095
Carotid resistive index (score)	0.79 (0.62–0.87)	0.76 (0.56–0.88)	0.022
Femoral resistive index (score)	0.85 (0.73–0.93)	0.86 (0.74–0.94)	0.499
Mean number of steps (*n*)	10,107 (4532–21,740)	6394 (1517–16,354)	<0.001
Tobacco smoking (%)	5.5	0	0.089
Alcohol drinking (%)	20	30.8	0.542

Note: Values are presented in frequencies or median (range). BMI (Body mass index). HOMA (Homeostatic Model Assessment) Significant differences in bold.

**Table 2:** Association between sports participation in early life, arterial thickness, and arterial resistive index.

	Sports Participation in Early Life	No Early Sports Participation	Wald	*p*	*r*	R^2^
Carotid intima-media thickness (mm)	0.64 ± 0.14	0.71 ± 0.21	6.54	0.011	−0.231	0.053
Femoral intima-media thickness (mm)	0.52 ± 0.10	0.60 ± 0.24	2.47	0.116	−0.119	0.014
Carotid resistive index (score)	0.78 ± 0.06	0.75 ± 0.06	0.40	0.529	0.053	0.003
Femoral resistive index (score)	0.85 ± 0.04	0.86 ± 0.04	3.18	0.075	−0.189	0.036

Notes. Values are presented in mean and standard deviation. Adjusted by chronological age, sex, tobacco smoking, alcohol consumption, C-reactive protein, HOMA index, mean blood pressure, body fat and actual physical activity.
